# Septic Arthritis of Hip Caused by *Salmonella typhi:* A Case Report

**DOI:** 10.1155/2012/464527

**Published:** 2012-09-09

**Authors:** M. Shanthi, Uma Sekar, K. S. Sridharan

**Affiliations:** ^1^Department of Microbiology, Sri Ramachandra Medical College & Research Institute, Sri Ramachandra University, Porur, Tamil Nadu 600116, Chennai, India; ^2^Department of Microbiology, Sri Ramachandra Laboratory Services, Sri Ramachandra Medical College & Research Institute, Sri Ramachandra University, Porur, Tamil Nadu 600116, Chennai, India

## Abstract

*Salmonella typhi* usually produces enteric fever and gastroenteritis. The infection may spread through blood stream and present as local suppurative lesions which may involve any site including the bone and joints. We report a case of septic arthritis of hip in a patient with systemic lupus erthematosis. The case is presented for its rarity and to highlight the atypical manifestations of *Salmonella typhi* in endemic regions.

## 1. Introduction 

Infection with *Salmonella typhi* can result in various presentations such as enteric fever, septicemia with and without local suppurative lesions, gastroenteritis, and the carrier state. The local suppurative lesions may involve any site in the body including the osteoarticular tissue. Though dissemination of infection can occur, septic arthritis due to *Salmonella typhi* is rare. Most patients with such metastatic infective lesions have underlying chronic disease or immunosuppressive state [1, 2]. We report a case of septic arthritis of hip due to *Salmonella typhi* in a patient with systemic lupus erythematosis (SLE) who was on long term steroid therapy.

## 2. Case 

**Figure 1:**
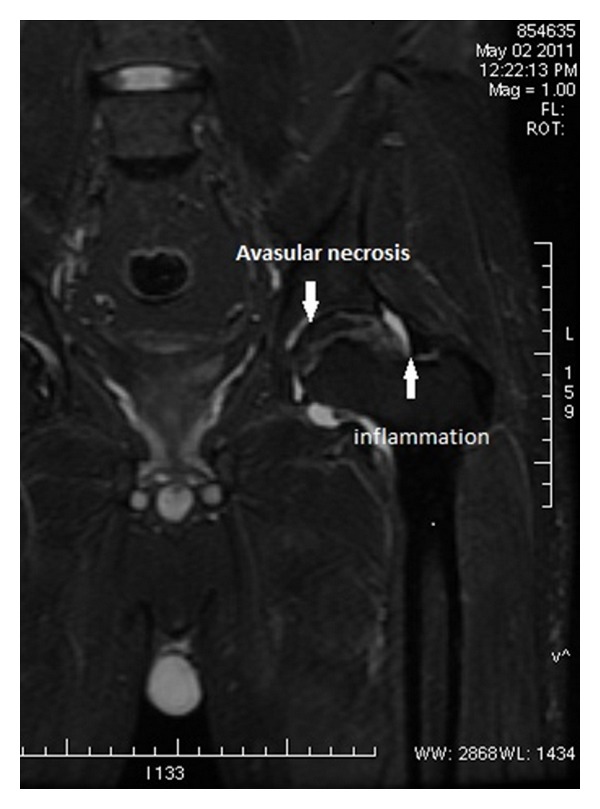
Magnetic resonance imaging (MRI) showing avascular necrosis and inflammation of the left hip.

A 40-year-old male patient was admitted to the orthopedic ward with chief complaints of pain, restriction of movements, and difficulty to bear weight on the left hip. The patient was a known case of SLE and on treatment with prednisolone 40 mg twice daily over the previous 6 years. In addition, he also had complaints of rashes over the trunk and pain in small joints of both hands for 2 months. The patient did not report any episode of fever over the preceding months. There was no history of trauma, prior surgery, or abdominal discomfort. There was no history of illness suggestive of typhoid fever in any of his family members. Local examination of the left hip region showed tenderness and the movements were painfully restricted. The laboratory findings were haemoglobin 9.8 gm%, white blood cell count 13,180 cells/cu.mm with neutrophilic leukocytosis. Peripheral smear showed microcytic normochromic anemia. Pus aspirated from the hip joint was inoculated in brain heart infusion broth, nutrient agar, 5% sheep blood agar, and MacConkey agar and incubated for 24 hours at 37°C. The gram stained smear of the pus showed plenty of polymorphonuclear leucocytes and gram negative bacilli. The culture yielded nonlactose fermenting colonies which was confirmed as *Salmonella typhi* by biochemical methods and serological typing. It was susceptible to ampicillin, trimethoprim-sulphamethoxazole, chloramphenicol, ciprofloxacin, cefotaxime, and ceftriaxone and resistant to nalidixic acid by disc diffusion method. The blood culture was found to be sterile. The Widal test was non-contributory. Fecal culture done on 3 consecutive days to detect carrier state or subclinical infection was negative. The radiograph and the magnetic resonance imaging of the left hip showed evidence of avascular necrosis of the neck of femur (Figure 1). In view of resistance to nalidixic acid, therapy with ciprofloxacin was not considered. Treatment with injection ceftriaxone 1 gram, intravenously twice daily was instituted and continued for 14 days. Surgical intervention was done (core decompression with free fibular grafting). The patient was explained about the guarded prognosis of the procedure. Histopathological examination of the biopsy specimen from the left neck and head of femur showed necrotic bone consistent with avascular necrosis. In view of the steroid induced avascular necrosis, patient was advised total hip replacement. However, the patient opted for discharge after relief from the pain.

## 3. Discussion

The genus *Salmonella* consists of a large heterogeneous group of gram negative bacilli that affect humans and animals. They are enteroinvasive and enteropathogenic organisms. Human beings are infected mainly by ingestion of food or water resulting in 4 types of clinical syndromes: enteric fever, septicemia with or without suppurative lesions, gastroenteritis, and carrier state [1]. 

Salmonellosis is endemic in developing countries. Though the most common manifestation of *Salmonella typhi* is fever, infection can spread through blood stream and present as focal lesions in any organ with or without suppuration. Localised infections at metastatic sites usually occurs in patients with preexisting diseases, such as hemoglobinopathies especially sickle cell disease [3, 4]. Osteoarticular infections occur commonly in the immunosuppressed [5]. In one study 36% had preexisting osteoarticular disease [6]. A common predisposing articular factor for Salmonella septic arthritis is avascular necrosis and the hip joint is the most frequently involved site [7].

Organisms which are commonly associated with septic arthritis include *Staphylococcus aureus, Haemophilus influenzae* type B, and* Streptococci*. *Salmonella* arthritis is infrequent and accounts for only 1% of all cases. It usually follows gastroenteritis and is caused most often by nontyphoidal *Salmonella*. Septic arthritis is an extremely rare complication of typhoidal *Salmonella* [1, 2]. This case was unique because, the Widal test was negative, the patient history did not reveal any significant fever in the preceding months, and the fecal culture to investigate carrier state or subclinical infection was noncontributory.

Early diagnosis, surgical intervention and administration of appropriate systemic antibiotics play a pivotal role in successful management. Chronic immunosuppression due to prolonged steroid therapy leads to depressed humoral immune response and this leads to insignificant widal titers [8]. Although infections can predispose to avascular necrosis [9], this patient having been on long term steroid therapy for a systemic collagen vascular disease, *Salmonella typhi *could have played a secondary contributory role.

## 4. Conclusion

Pyogenic joint infections caused by *Salmonella typhi* are rare despite its endemic occurrence in India. This case is reported to highlight the unusual presentation of *Salmonella typhi.* Conventional diagnostic methods including Widal and blood culture may not be useful for diagnosis. In chronic immunosuppressed individuals, typical history of fever is lacking. Isolation of *Salmonella typhi* from the joint remains the gold standard. Physicians should be aware of the rare manifestations of *Salmonella typhi* infections especially in endemic areas. Apart from surgical intervention, definitive early diagnosis of the infection and institution of appropriate antibiotics can minimize the damage to the affected joint.
